# Resilience of *Zostera muelleri* seagrass to small-scale disturbances: the relative importance of asexual versus sexual recovery

**DOI:** 10.1002/ece3.933

**Published:** 2014-01-21

**Authors:** Peter I Macreadie, Paul H York, Craig DH Sherman

**Affiliations:** 1 Centre for Environmental Sustainability (CEnS), School of the Environment, University of TechnologySydney, New South Wales, 2007, Australia; 2 Plant Functional Biology and Climate Change Cluster (C3), School of the Environment, University of TechnologySydney, New South Wales, 2007, Australia; 3 School of Life and Environmental Sciences, Centre for Integrative Ecology, Deakin UniversityVictoria, 3216, Australia

**Keywords:** Disturbance, genotypic diversity, recovery, resilience, seagrass, Zostera

## Abstract

Resilience is the ability of an ecosystem to recover from disturbance without loss of essential function. Seagrass ecosystems are key marine and estuarine habitats that are under threat from a variety of natural and anthropogenic disturbances. The ability of these ecosystems to recovery from disturbance will to a large extent depend on the internsity and scale of the disturbance, and the relative importance of sexual versus asexual reproduction within populations. Here, we investigated the resilience of *Zostera muelleri* seagrass (Syn. *Zostera capricorni*) to small-scale disturbances at four locations in Lake Macquarie – Australia's largest coastal lake – and monitored recovery over a 65-week period. Resilience of *Z. muelleri* varied significantly with disturbance intensity; *Z. muelleri* recovered rapidly (within 2 weeks) from low-intensity disturbance (shoot loss), and rates of recovery appeared related to initial shoot length. Recovery via rhizome encroachment (asexual regeneration) from high-intensity disturbance (loss of entire plant) varied among locations, ranging from 18-35 weeks, whereas the ability to recover was apparently lost (at least within the time frame of this study) when recovery depended on sexual regeneration, suggesting that seeds do not provide a mechanism of recovery against intense small-scale disturbances. The lack of sexual recruits into disturbed sites is surprising as our initial surveys of genotypic diversity (using nine polymorphic microsatellite loci) at these location indicate that populations are maintained by a mix of sexual and asexual reproduction (genotypic diversity [*R*] varied from 0.24 to 0.44), and populations consisted of a mosaic of genotypes with on average 3.6 unique multilocus genotypes per 300 mm diameter plot. We therefore conclude that *Z. muelleri* populations within Lake Macquarie rely on clonal growth to recover from small-scale disturbances and that ongoing sexual recruitment by seeds into established seagrass beds (as opposed to bare areas arising from disturbance) must be the mechanism responsible for maintaining the observed mixed genetic composition of *Z. muelleri* seagrass meadows.

## Background

There is still major uncertainty about how climate change will affect marine ecosystems, largely because of a lack of understanding of the processes that provide insurance against environmental change, that is ecosystem resilience. In a broad sense, resilience refers to the capacity of ecosystems to cope with disturbance, without switching to an alternative (and undesirable) stable state, sometimes referred to as a “phase or regime shift.” Many ecologists believe that if the factors that mediate resilience for a given ecosystem can be predicted, monitored, and modified, then desired ecosystem states could be maintained in the face of increasing environmental change (Folke et al. 2004).

There is currently a global push toward understanding the mechanisms that underpin resilience in seagrass ecosystems for two reasons. First, because of their global importance, seagrasses stabilize shorelines and prevent coastal erosion (Bos et al. 2007); they play a key role in nutrient cycling [worth US$19K ha-1 year-1; (Costanza et al. 1997)]; they provide critical habitat for fish, bird, and invertebrates (Heck et al. 2003; Hughes et al. 2009); and they are one of the earth's most powerful carbon sinks (McLeod et al. 2011; Fourqurean et al. 2012; Macreadie et al. 2013). Second, because they are currently facing a global crisis (Orth et al. 2006); 29% of the world's seagrasses have disappeared (Waycott et al. 2009), and 14% of all seagrass species are at risk of extinction (Short et al. 2011).

The alternative stable state of seagrasses is typically represented as an environment dominated by bare sediment or ephemeral algae, whereby sediment stability and particle trapping from the water column are no longer maintained, thereby creating a feedback loop that prevents establishment of seagrass roots and a low-quality light environment (van der Heide et al. 2007; Hendriks et al. 2008). Alternative stable states in seagrass ecosystems are generally thought to be caused by large-scale disturbance events (e.g., eutrophication); however, small-scale disturbances that create gaps in seagrass meadows (e.g., anchor and boat damage, grazing, and storms) can also cause alternative stable states (Meehan and West 2000) and autocatalytic decline (Larkum and West 1982), yet they have received little attention, and they are becoming increasingly common in urbanized areas of the coast.

Seagrass recovery from fine-scale disturbance can occur through both sexual and asexual mechanisms, the importance of which will depend to a large extent on the levels and distribution of genotypic diversity within a population, the frequency of disturbance events, and the frequency of sexual reproduction (Eriksson 1993; Reusch et al. 2005; Reusch 2006; Becheler et al. 2010). In mixed mating systems where sexual reproduction is frequent, disturbance is predicted to increase and maintain high levels of genotypic diversity (Williams 1975; Bell 1982; Jackson et al. 1985). This is because the opening of new space should allow for the recruitment of new sexual recruits that would otherwise be competitively excluded by established adults. Disturbance is also predicted to enhance genotypic diversity by preventing competitively superior genotypes from dominating spatially. In contrast, populations with low levels of genotypic diversity and/or sexual events are more likely to recover from disturbance through the asexual proliferation of established genotypes.

Studies on the relative importance of sexual versus asexual mechanisms of recovery by seagrass following disturbance have reported varying results, with some studies showing that asexual recolonization through rhizome growth is the dominant mechanism of recovery (Larkum and West 1982; Rasheed 1999; Meehan and West 2000; Jarvis and Moore 2010), while other studies have highlighted the importance of recovery from sexual recruits (Plus et al. 2003; Reusch 2006; Becheler et al. 2010). These contrasting results are likely to result to some extent from differences in the levels of genotypic diversity within populations. While most studies have not measured levels of genotypic diversity within populations prior to disturbance, assessment of the underlying levels of genotypic diversity prior to disturbance is crucial for predicting and interpreting patterns of recovery after a disturbance. This has been demonstrated experimentally by Reusch (2006) who showed that recolonization of disturbed sites in the seagrass *Zostera marina* was strongly correlated with initial levels of standing genotypic diversity within those sites. Thus, those sites with high levels of genotypic diversity prior to the disturbance had the greatest number of new genotypes recruiting to those during the monitored recovery period. It is therefore important when carrying out disturbance/recovery experiments to assess levels of standing genotypic diversity within seagrass meadows as this potentially allows for a better understanding of the capacity for sexual versus asexual recruitment after a disturbance. This information is also important when carrying out disturbance experiments across different geographical locations (such as this study) where variation in the levels of genotypic diversity among sites may result in different mechanisms of recovery.

Using disturbance/recovery experiments, we investigated factors that mediate resilience of *Zostera muelleri* (Syn. *Z. capricorni*) to small-scale disturbances in Australia's largest coastal lake, the Lake Macquarie estuary. We were specifically interested in how resilience varies with intensity of disturbance (above- and below-ground removal of plant material vs. above-ground removal only), the mode of regeneration (sexual – seeds vs. asexual – vegetative, clonal growth), and how resilience varies locally (within locations) and regionally (among locations). We also measured several environmental characteristics (e.g., temperature, sediment grain size and organic content, and infaunal abundance and species richness) to explain potential differences in resilience among locations. Furthermore, we assessed levels of genotypic diversity within and among locations to determine the relative importance of sexual and asexual reproduction to maintaining populations and therefore the capacity for both modes of reproduction to contribute to recovery post-disturbance.

The study took place in a region of Australia's east coast that has suffered major declines in seagrass cover in recent years [˜50% in New South Wales estuaries; Walker and McComb (1992)], particularly *Z. muelleri*, which is the most widespread species in this region. Rasheed (Rasheed 1999, 2004) has previously demonstrated that asexual regeneration is the most important recovery mechanism for this species in the tropical zone, but such information for temperate populations is lacking. *Z. muelleri* belongs to the Zosteraceae family, which is the dominant family in temperate latitudes, and is regarded as a globally significant congeneric species. We predicted that resilience of *Z. muelleri* will decrease with increasing disturbance intensity, and that the recovery via asexual regeneration will be faster than sexual regeneration.

## Methods

### Study location

This study was conducted in Lake Macquarie; Australia's largest coastal saltwater lake. The Lake covers an area of 110 km^2^ and is situated 130 km north of the city of Sydney, on the east coast of Australia. The Lake has an irregular shoreline with many bays and promontories along its 170 km perimeter. It has an average water depth of ˜8 m and is connected to the ocean via a constricted entrance that limits tidal variation. Seagrass is abundant in the Lake, although restoration efforts and dedicated management efforts have been necessary for reverting declines in seagrass cover due to urbanization around the Lake over the past few decades. The Lake now contains one of the largest seagrass populations on the New South Wales coast, representing 10% of the total seagrass area in NSW.

The main species of seagrass in Lake Macquarie are *Zostera muelleri*, *Posidonia australis*, *Heterozostera nigricaulis*, *Halophila ovalis* and *Halophila decipiens*. This study focused on *Z. muelleri*; the most abundant species within the Lake. We selected four study locations within the Lake: Sunshine (33°06′29.82″S, 151°33′52.31″E), Valentine (32°59′46.04″S, 151°37′56.08″E), Wangi (33°03′51.54″S, 151°34′59.70″E), and Point Wolstoncroft (33°07′07.61″S, 151°35′20.95″E). Each location contained relatively continuous meadows of subtidal (˜0.2–1.5 m below mean low water spring; MLWS) seagrass running parallel to the shore.

### Experimental design

Disturbance/recovery experiments were adapted to measure resilience. Disturbance in seagrass ecosystems typically manifests in the form of habitat loss; thus, experimental removal of habitat was used to represent disturbance. Resilience (which includes the ability of a system to recovery rapidly from loss of structure or function) was measured by the rate of seagrass recovery (i.e., time taken for % cover to return to background levels) following habitat loss. We used a factorial design with three main factors: disturbance treatment, time since disturbance, and location. Locations (fixed factor) are described previously, and time since disturbance (repeated measures) simply represented the times that the different disturbance treatments were sampled after they were established (October 20, 2010): 0, 2, 6, 12, 18, 36, and 65 weeks.

**Figure 1:**
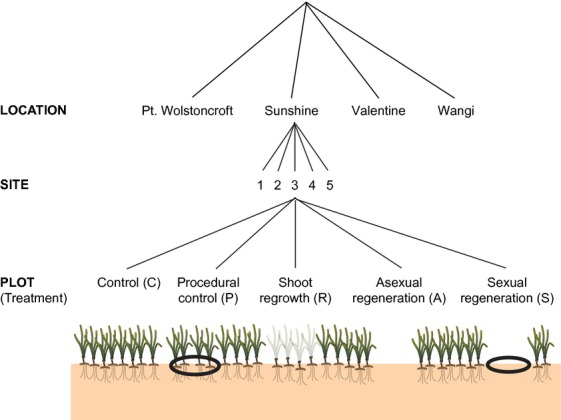
Hierarchical (fully crossed) experimental design. Each of the four locations (all within Lake Macquarie; NSW, Australia) had five sites, and each site had five experimental treatments assigned to plots. Experimental treatments: control (C) – seagrass left untouched; procedural control (P) – seagrass with a border (shown as a black ring); shoot regrowth (R) – seagrass with above-ground plant material removed; asexual regeneration (A) – seagrass with above- and below-ground plant material removed; and sexual regeneration (S) – seagrass with above- and below-ground plant material removed and a border emplaced to prevent rhizome encroachment. Diagram produced using the Integration and Application Network (IAN), University of Maryland Center for Environmental Science, Cambridge, Maryland.

The five different disturbance treatments were (Fig. 1): control (C) – seagrass left untouched; procedural control (P) – seagrass with a border; shoot regrowth (R) – seagrass with above-ground plant material removed; asexual regeneration (A) – seagrass with above- and below-ground plant material removed; and sexual regeneration (S) – seagrass with above- and below-ground plant material removed and a border emplaced to prevent rhizome encroachment. The above-ground removal only represents a low-intensity disturbance (e.g., herbivore grazing), whereas the above- and below-ground removal represent a high-intensity disturbance, typical of mechanical damage (e.g., boat propeller scarring).

At the time disturbances were applied, the average seagrass length across sites was 17 ± 2 cm (mean ± SE), and the average seagrass density was 482 ± 22 shoots per m^2^. The plot area used for each disturbance treatment was a 300 mm diameter circle (area = 0.07 m^2^). Similar sized disturbances have been shown to influence the rate and mode of *Z. muelleri* recovery (Rasheed 1999, 2004). To prevent recolonization from disturbance, we inserted borders (made from round PVC piping, 300 mm diameter) into disturbance plots to a depth of 95 mm, leaving 5 mm of border exposed above the sediment surface. Borders prevented recolonization of disturbance plots from the surrounding meadow by acting as a barrier against rhizome encroachment – that is vegetative regrowth into disturbed plots was prevented by borders. Plots were inspected at each sampling occasion for rhizomes growing over the top of borders into plots. On rare occasions, where rhizome jumping had occurred (as detected by tracing plants within plots to their origin outside of plots), these plants were removed.

At each location, a total of 5 “sites” (˜2 m x 2 m) were established at a distance of at least 20 m apart and at a depth of ˜0.5 m MLWS with a replicate of each disturbance treatment haphazardly placed within (Fig. 1). Recovery following high-intensity disturbance will rely on outside sources for recolonization (e.g., seeds, encroachment of rhizomes from the surrounding meadow, deposition of drifting whole plants), whereas recovery from low-intensity disturbance should occur through regrowth from existing rhizome material. Therefore, we predicted that recovery times would be significantly faster in low-intensity disturbance treatments than high-intensity treatments.

### Sampling

Experimental treatments were sampled 0, 2, 6, 12, 18, 36, and 65 weeks after their establishment (October 20, 2010). Sampling involved visually estimating each replicate for % cover [using Seagrass Watch standard protocols, which involved two observers and use of a % cover photograph standard; McKenzie et al. 2003], the presence of flora or fauna, seagrass canopy height (6 haphazard measurements per replicate), the approximate amount of epiphyte cover on seagrass blades (low, medium, or high), and density of shoots (controls only). Photographs of each replicate were taken for reference purposes. Plastic star pickets were used to mark each plot. To characterize locations, we measured wet bulk density, and organic matter, and mean shoot length at the start of the experiment.

### Genetic sampling and genotyping

Levels of standing genotypic diversity within each location were assessed in order to establish the relative importance of sexual and asexual reproduction in maintaining populations. Within each location, genetic samples of *Z. muelleri* were collected by randomly selecting 8 shoots from each of three 300-mm-diameter plots within each of the five sites used for the resilience experiments. Thus, for each location, a total of 120 samples were collected for genetic analysis (480 samples in total across the four locations). Samples were desiccated by storing them on silica crystals. Lyophilized leaf tissue (˜10 mg per sample) was first frozen in liquid nitrogen, pulverized in a TissueLyser II, and DNA extracted using DNeasy plant kits (QIAGEN, Germantown, MD), following the manufacturer's instructions. Nine polymorphic microsatellite loci [ZosNSW02, ZosNSW15, ZosNSW18, ZosNSW19, ZosNSW23, ZosNSW25, ZosNSW29, ZosNSW38, ZosNSW46; Sherman et al. 2012)] were amplified using polymerase chain reactions (PCRs) conducted in 11 *μ*L volumes containing; 10 ng of genomic DNA; 5 *μ*L PCR Master Mix (Qiagen) and 4 *μ*L primer multiplex (0.26 *μ*M of each forward primer and fluorescent dye, 0.13 *μ*M of reverse primer). Thermal cycling condition used a touchdown program with an initial hot start at 94°C for 15 min; five cycles of 94°C for 45 sec, 65°C for 45 sec, 72°C for 45 sec; five cycles of 94°C for 45 sec, 60°C for 45 sec, 72°C for 45 sec; 10 cycles of 94°C for 45 sec, 57°C for 45 sec, 72°C for 45 sec; 20 cycles of 94°C for 45 sec, 55°C for 45 sec, 72°C for 45 sec; and a final elongation at 72°C for 15 min. PCR products were electrophoresed using an ABI 3130xl Genetic Analyzer, incorporating LIZ 500 (-250) size standard (Applied Biosystems) and alleles were scored using GeneMapper, v3.7 (Applied Biosystems).

### Statistical analyses

Data were analyzed using univariate (SPSS) statistical techniques. The main response variable of interest was the percent cover of seagrass. Percent cover was analyzed using a repeated measures ANOVA, with location and treatment as between subject factors, and time since disturbance as the within subjects factor. The degrees of freedom for the within subject factors were adjusted using the Greenhouse-Geiger correction to meet assumptions of sphericity. Significant main effects were analyzed further with post hoc Student–Neuman–Keuls (SNK) tests to identify significant differences among means.

We tested the power of the genetic marker system to detect unique multilocus genotypes (G) by calculating the probability of identity, P_ID_, for increasing locus combinations (Waits et al. 2001) using the program GenAlex (V6) (Peakall and Smouse 2006). P_ID_ calculates the probability that two individuals drawn at random within a population will have the same multilocus genotype and can be used to estimate the expected number of individuals with the same multilocus genotype within samples (calculated as *P*_ID_ × sample size). Levels of genotypic diversity were expressed as *R*, where *R *= (*G* − 1)/(*N* − 1) (Dorken and Eckert 2001).

## Results

### Genotypic diversity

The number of alleles detected at each locus varied from 2 to 13, with a mean of 6.8 ± 1.5 (SE) alleles per locus over all loci and locations. This provided a high level of power in identifying distinct multilocus genotypes within our samples. The probability of identity was low for all loci combined (P_ID_ < 0.001, Table 1) indicating that the probability of the same multilocus genotype arising more than once through sexual reproduction is extremely low. Thus, the number of individuals that are expected to have the same multilocus genotype was always less than 1 (Table 2). Samples with identical multilocus genotypes across all nine microsatellite loci were therefore regarded as belonging to the same clone (i.e., ramets).

**Table 1:** The probability of identity for increasing seagrass locus combinations.

Population	*N*	*ZosNSW25*	*+ZosNSW19*	*+ZosNSW29*	*+ZosNSW02*	*+ZosNSW38*	*+ZosNSW46*	*+ZosNSW18*	*+ZosNSW23*	*+ZosNSW15*
Point Wolstoncroft	120	1.000	0.127	0.047	0.047	0.009	0.001	0.001	0.001	<0.001
Sunshine	119	1.000	0.182	0.068	0.068	0.017	0.001	0.001	0.001	<0.001
Valentine	120	0.154	0.008	0.002	0.001	<0.001	<0.001	<0.001	<0.001	<0.001
Wangi	118	0.150	0.010	0.003	0.001	<0.001	<0.001	<0.001	<0.001	<0.001

Calculated for the seagrass *Zostera muelleri* from four locations in NSW, Australia.

**Table 2:** Expected number of individuals with the same seagrass multilocus genotype.

Population	*N*	*ZosNSW25*	*+ZosNSW19*	*+ZosNSW29*	*+ZosNSW02*	*+ZosNSW38*	*+ZosNSW46*	*+ZosNSW18*	*+ZosNSW23*	*+ZosNSW15*
Point Wolstoncroft	120	120.00	15.24	5.68	5.68	1.05	0.07	0.07	0.07	0.04
Sunshine	119	119.00	21.62	8.12	8.12	2.06	0.07	0.07	0.07	0.03
Valentine	120	18.53	0.98	0.18	0.08	0.02	<0.01	<0.01	<0.01	<0.01
Wangi	118	17.72	1.24	0.33	0.13	0.03	<0.01	<0.01	<0.01	<0.01

Calculated as respective probability from Table 1 x population size) for increasing locus combinations for the seagrass *Zostera muelleri* from four locations in NSW, Australia.

**Figure 2:**
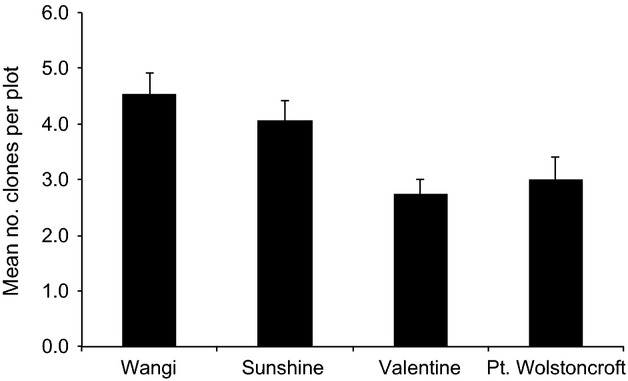
Clonal diversity of *Zostera muelleri*. Mean number of clones detected within 300-mm-diameter plots across four locations within Lake Macquarie, NSW, Australia. Within each plot, 8 samples were analyzed with 5 plots sampled across 5 sites within each location (total number of samples per location = 120).

Levels of genotypic diversity (*R*) indicated that the relative importance of sexual and asexual reproduction varied across locations. Valentine and Point Wolstoncroft displayed the lowest levels of genotypic diversity (*R *=* *0.24 and *R *=* *0.27 respectively), while Wangi and Sunshine displayed almost twice the amount of genotypic diversity seen at other locations (*R *=* *0.44 at both locations). At a fine-spatial scale (i.e., within 300 mm diameter plots), our sampling revealed a mosaic of genotypes. The number of unique multilocus genotypes detected within our samples of eight shoots per 300 mm diameter plot varied from 1 to 8 genotypes per plot, with a mean of 3.6 ± 0.2 (SE) genotypes detected per plot across all locations. The mean number of genotypes per plot varied significantly among locations, with the fewest genotypes per plot detected at Valentine (2.7 ± 0.27 (SE) genotypes per plot), while Wangi displayed the greatest number of genotypes per plot (with 4.5 ± 0.39 [SE] genotypes per plot; Fig. 2). A GLM analysis revealed that Wangi had a significantly higher number of clones per plot compared to Valentine and Point Wolstoncroft, but not Sunshine (*F*_3, 59_ = 5.72, *P *=* *0.002; Fig. 2), while there was no difference in the number of clones per plot between any of the three other locations (Fig. 2).

### Resilience experiments

**Figure 3:**
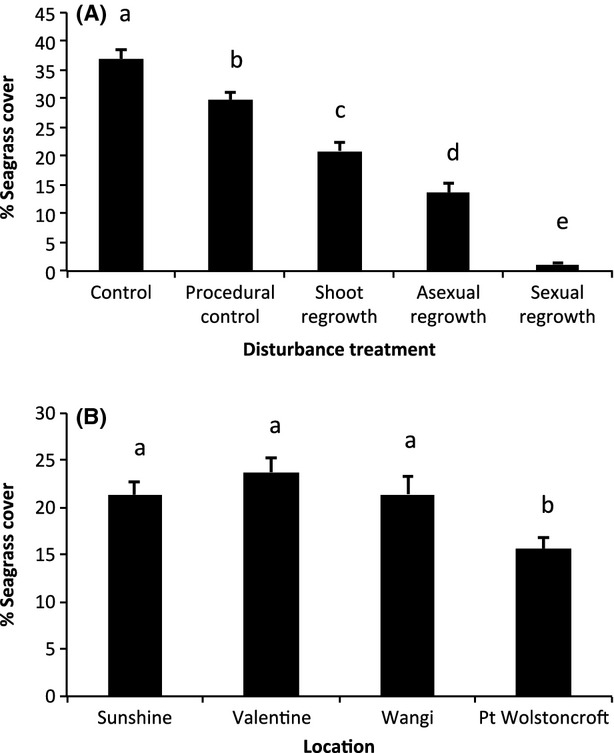
Differences in seagrass percent cover. Differences in the % seagrass (mean ± SE) cover among (A) disturbance treatment (*n *=* *140; location and time since disturbance pooled) and (B) locations (*n *=* *175; disturbance treatment and time since disturbance pooled). Post hoc SNK tests were used to determine which disturbance treatments and locations differed from each other; bars that have same letters above are not significantly different (*P *>* *0.05).

**Figure 4:**
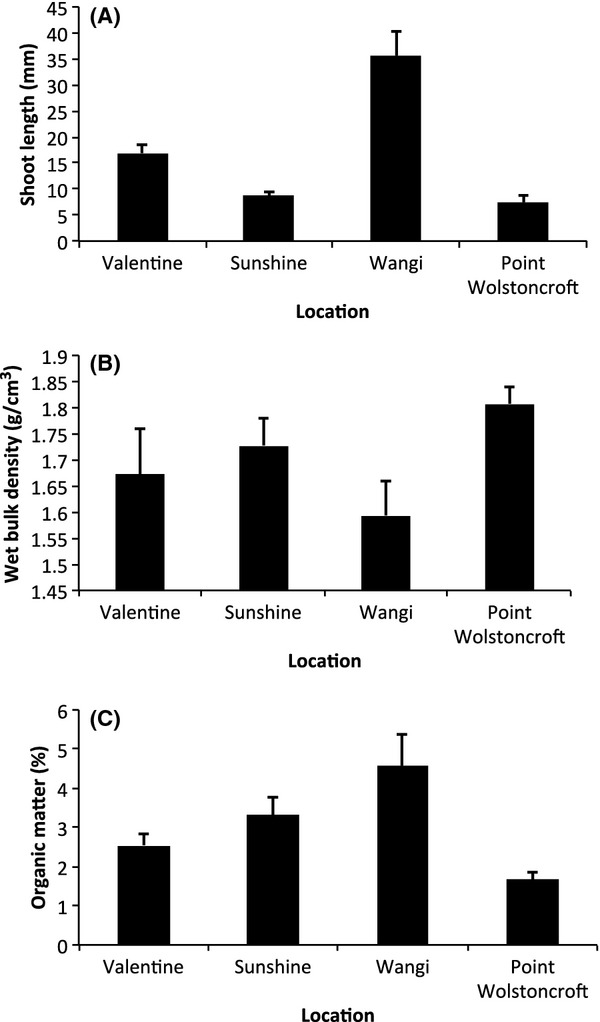
Location characterization. Variation (mean ± SE) in (A) shoot density (*n *=* *5); (B) wet bulk density (*n *=* *3); and (C) organic matter content (*n *=* *3) among locations.

We detected significant differences in percentage seagrass cover for all the main effects and interactions, with the exception of disturbance treatment × location, which was marginally non-significant (Table 3). Post hoc tests showed that each disturbance treatment differed from one another in their percentage seagrass cover (Fig. 3A): controls had the highest seagrass cover (37%), followed by the procedural control (30%), regrowth (21%), asexual regeneration (14%), and sexual regeneration (1%). The 1% recovery in sexual regeneration may actually be the result of rhizomes jumping over borders; indeed, we did observe rhizomes jumping borders throughout the experiment, but the reduced frequency of sampling toward the end of the experiment made it difficult to trace the rhizomes of plants that had established within plots to the surrounding meadow. Sunshine, Valentine, and Wangi locations had similar percentage seagrass cover (21%, 24%, and 21%, respectively; Fig. 3B), whereas the percent seagrass cover at Point Wolstoncroft was significantly lower (16%; Fig. 3B). Point Wolstoncroft had the shortest shoot length (Fig. 4A), highest wet bulk density (Fig. 4B), and lowest sediment organic matter content (Fig. 4C).

**Table 3:** Comparing percent cover of seagrass among disturbance treatment (*D*), location (*L*), and time since disturbance (*T*).

Source	df	Mean Square	*F*	*P*-value
*Within subjects*				
Time since disturbance (*T*)	2.86	12776.5	49.76	**<0.001**
*D* x *T*	11.44	1752.1	6.82	**<0.001**
*L* × *T*	8.58	777.7	3.03	**0.002**
*D* × *L* × *T*	34.31	403.3	1.57	**0.029**
Error (*T*)	217.27	256.8		
*Between subjects*				
Disturbance treatment (*D*)	4	25865.4	64.57	**<0.001**
Location (*L*)	3	1708.5	4.27	**0.008**
*D* × *L*	12	677.5	1.69	0.085
Error	76	400.6		

Repeated measures ANOVA results. *D* and *L* were the between subject factors, and *T* was the within subject factor. The degrees of freedom for the within subject factors were adjusted using the Greenhouse-Geisser correction to meet assumptions of sphericity. Bold value represents P < 0.05.

**Figure 5:**
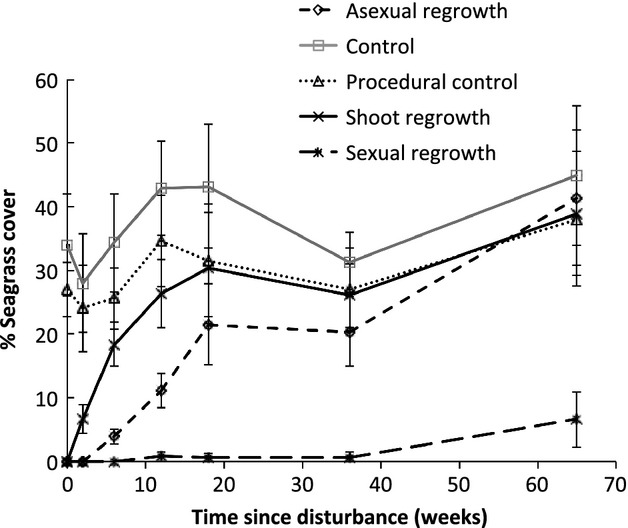
Changes in seagrass percent cover through time. Changes in % cover (mean ± SE, *n *=* *5) of seagrass among experimental treatments over the 15-month experimental period averaged across all locations.

Trends through time in percentage seagrass cover for each location and treatment are shown in Figure 5. The disturbance treatments all exhibited a similar pattern at each location; within 2 weeks, regrowth had begun to recover. Regrowth recovered completely at all locations, although the rate of recovery varied among locations and appeared to be related to the initial length of the seagrass – that is rates of recovery increased with the initial length of the seagrass (Fig. 4A). For asexual regeneration, recovery took place by week 18 at Valentine and Sunshine, 36 weeks for Wangi, and at Point Wolstoncroft, there was partial recovery by 18 weeks, followed by a decline to near-zero at 36 weeks, and then near-complete recovery at 65 weeks. Sexual regeneration did not completely recovery at any location.

**Figure 6:**
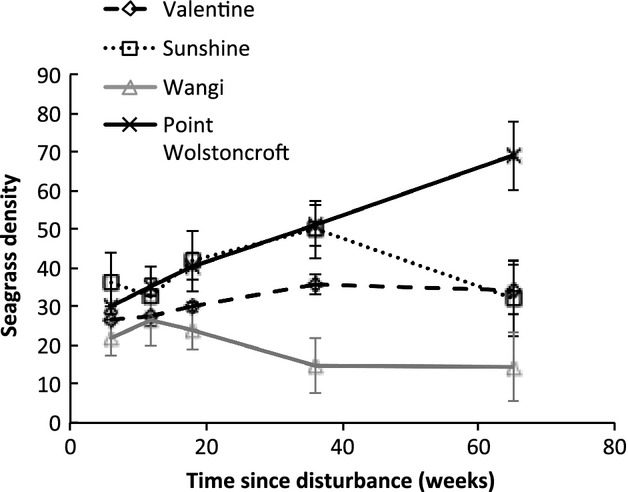
Variation in seagrass density through time. Changes in seagrass density (mean ± SE, *n *=* *5) in controls among study locations over the study period.

Percentage seagrass cover in control and procedural control treatments varied with season, with higher percentage seagrass cover during warmer months, and lower percentage cover during cooler months. Contrastingly, seagrass density did not vary with season (Fig. 6). Seagrass density at Point Wolstoncroft increased throughout the experiment, more than doubling the number of shoots in control plots during the 65-week monitoring period. Seagrass density at Sunshine and Valentine remained more or less constant, and mean seagrass density at Wangi decreased slightly.

**Figure 7:**
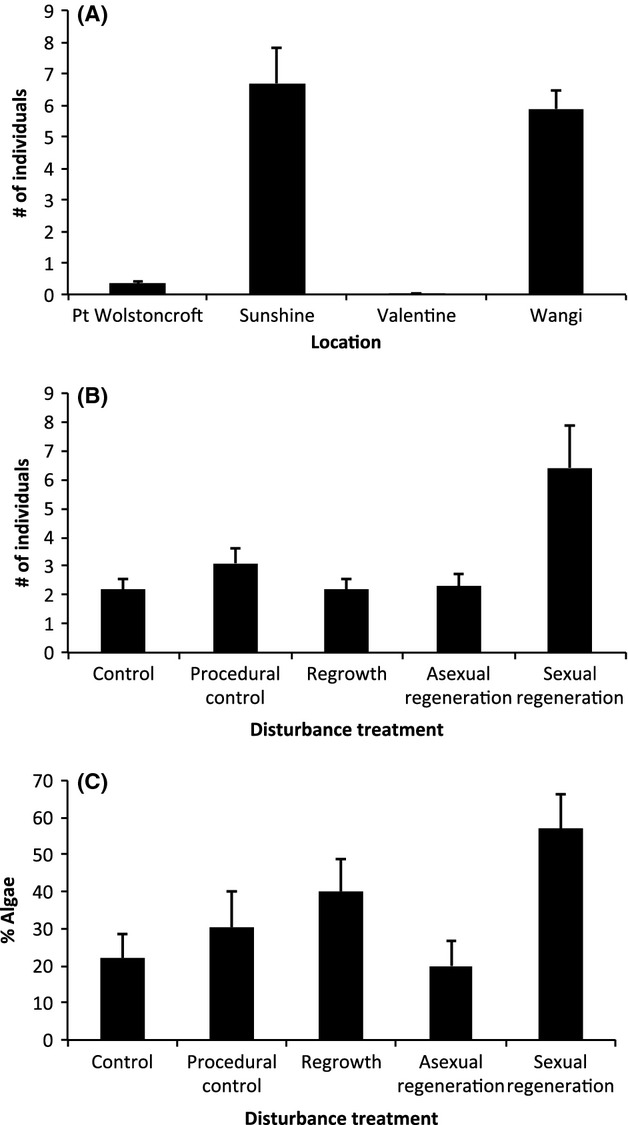
Faunal densities and algal presence within plots. Variability in the abundance (mean ± SE) of individual animals recorded (A) at four locations within Lake Macquarie (*n *=* *100) in (B) experimental treatments (*n *=* *125; control – C; procedural control – P; shoot regrowth – R; asexual regeneration – A; and sexual regeneration – S); and (C) differences in the % cover of algae (mean ± SE, *n *=* *20) among disturbance treatment at Wangi.

The abundance of individual animals in disturbance treatments differed among locations (Fig. 7A); of the 1618 individuals recorded, 52% were from Sunshine, 45% from Wangi, 3% from Point Wolstoncroft, and <1% from Valentine. Abundance also differed among disturbance treatments (Fig. 7B), with sexual regeneration having much higher abundances of individual animals. Numerically dominant animals were *Batillaria australis* (gastropod; 62%), *Pagurus sinuatus* (hermit crab; 33%), and *Anadara trapezia* (bivalve; 3%). The presence of algae in disturbance treatments was rare. The only location with a consistent presence of algae was Wangi (Fig. 7C).

## Discussion

We found that *Z. muelleri* is resilient to both low- and high-intensity disturbances when asexual regeneration is possible, but that resilience is apparently lost (at least within the time frame of the study; 65 weeks) when sexual regeneration is the only available mode of recovery. These results indicate that clonal growth is the critical mode of recovery from small-scale disturbances and that despite the potential for both sexual and asexual reproduction within these locations, the recovery of disturbed plots via sexual propagules occurs rarely. Thus, seed production does not appear to provide resilience against small-scale disturbances; a conclusion that is supported by several other studies (e.g., Rasheed 1999; Boese et al. 2009; Preen 1995; Williams 1990; Olesen et al. 2004).

There were several environmental variables that might have mediated the resilience of *Z. muelleri* to disturbance. The first was the presence of animals. Disturbance treatments that recovered had significantly lower abundances of animals (gastropods, bivalves, and hermit crabs) than the disturbance treatment that did not recover (sexual regeneration). It is therefore possible that the presence of animals prevent recovery in disturbed areas of seagrass, which has been previously reported for herbaceous animals (Sumoski and Orth 2012), but to our knowledge, this phenomenon has not yet been reported for nonherbaceous animals (detritivores in this case). A possible mechanism whereby animals could prevent sexual regeneration would be if they reworked sediment and caused either loss of seeds or an unsuitable environment for seedling establishment (Dumbauld and Wyllie-Echeverria 2003).

Another notable environmental variable that was relevant to the Wangi location, which might have prevented recovery in the sexual regeneration treatment, was eutrophication. Seagrasses at Wangi were exposed to storm-water runoff that drained directly from a pipe in the middle of the location, which explains the low light levels, high organic matter loads, high epiphyte load, and the presence of large benthic macroalgal mats at this location. Consequently, the seagrasses at this location had low densities and high shoot lengths. Furthermore, we suggest that benthic algal mats might have prevented seed establishment and germination. Drifting algae can cause loss of seedlings through physical disturbance (Valdemarsen et al. 2010), and algae can reduce survival of seed-producing shoots and cause suffocation due to light limitation and unfavorable biochemical conditions (Bintz and Nixon 2001; van Katwijk et al. 2010).

Fluctuations in percentage seagrass cover in control and procedural control appeared to match seasonal temperature variation, with higher percentage seagrass cover during warmer months, and lower percent cover during cooler months. The experiment was established during the middle of the Austral spring, which is when the seagrasses were coming out of their winter scenescent phase, and moving into a growth-reproduction phase. This same pattern does not exist for the density profiles, suggesting that density is not a good indicator of seagrass productivity. The reason for this is because percent cover changes with temperature throughout the year as seagrasses move through growth scenescence cycles, which are likely to affect the above-ground plant material such as the number and size of leaves per shoot rather than the number of shoots (density).

We found no relationship between levels of genotypic diversity at a location and the relative importance of sexual versus asexual reproduction to recovery of disturbed plots, or the rate of recovery of plots. Recovery by seed was not confirmed within any locations, even though we found significant differences in levels of genotypic diversity between locations. Thus, recovery to fine-scale disturbance seems to be driven primarily by asexual rhizome growth in this system. Studies on the effect of genotypic diversity on resilience and the rate of recovery to disturbance have been carried out for the closely related seagrass *Z. marina* (Hughes and Stachowicz 2004). The study by Hughes and Stachowicz (Hughes and Stachowicz 2004) used experimental manipulations of genotypic diversity of constructed plots to test for resistance to disturbance and time to recovery after a disturbance. While their study was not designed to test the relative importance of sexual versus asexual reproduction to recovery, their study did show that plots with higher genotypic diversity had enhanced community resistance to some disturbances (e.g., grazing by geese) and that genotypically more diverse plots has quicker recovery times (Hughes and Stachowicz 2004).

While variation in genotypic diversity does not seem to influence the mechanism of recovery of seagrass beds in this system, there does appear to be a relationship between genotypic diversity and animal abundance. There were higher levels of animal abundance at the two most genotypically diverse locations (Sunshine and Wangi, *R *=* *0.44 at both locations), which together accounted for 97% of all animals recorded. The two genotypically poorer locations (Valentine and Point Wolstoncroft, *R *=* *0.24 and 0.27, respectively) only accounted for 3% of all animals recorded. Thus, there appears to be a relationship between levels of genotypic diversity and animal abundance, although it remains unclear if this affects resilience and recovery of the ecosystem under different disturbance regimes.

The results from our genotypic surveys also suggest that fine-scale disturbance is not likely to be the main mechanism driving the mixed genotypic composition observed within our plots. Fine-scale disturbance does not appear to provide new space for the recruitment of sexual propagules, as suggested by Becheler et al. (2010). Instead, the mixing of genotypes in seagrass meadows at fine-spatial scales observed here is likely to result from a mixture of ongoing sexual recruitment by seeds into established seagrass beds, with subsequent spreading and intermingling of rhizomes from different clones over small spatial scales. The frequency and timing of sexual events in these populations remain unknown.

It is worth noting that the seagrasses we studied in Lake Macquarie might be subjected to slight artificial warming from two coal-fired power stations (Eraring and Vales Point) that release hot effluent into the Lake. Higher temperatures can affect seagrasses' sexual reproduction (Cabaco and Santos 2010), shoot density (Ehlers and Worm 2008; Diaz-Almela et al. 2009), seed viability (Kishima et al. 2011), and, concomitantly, could affect their resilience to disturbance. Indeed, the temperatures reached during the peak of summer (up to 33°C in January and February) have been shown to cause metabolic imbalances (reductions in photosynthesis and increases in leaf respiration) in this species (Collier et al. 2011). However, these high temperatures were only experienced very briefly (several hours at most), and it is difficult to know what effect these “pulses” might have – most research on thresholds have been performed with temperature held constant, but see Campbell et al. (2008), which are less environmentally relevant.

We did not observe any flowering or seed production during the course of the experiment, even though flowers and seeds have been observed at these same locations outside this experiment. It is possible that the timing of our sampling did not coincide with reproductive events, or, alternatively, it may be that there was no flowering and seed production during the experiment (>1 year duration). We did not measure seed banks, but the lack of recovery via seeds suggests that they did not exist, or the conditions were not right for germination. Seed banks have been shown to be an important mode of recovery for seagrasses from a range of large-scale disturbances, including: high water temperatures (Jarvis and Moore 2010), storm disturbance (Hammerstrom et al. 2006), and anoxia (Plus et al. 2003). By comparison, there is little explicit evidence that seed banks play an important role in recovery from small-scale disturbances, despite some genetic studies suggesting otherwise (Zipperle et al. 2009; Becheler et al. 2010).

The use of borders to manipulate seagrass regeneration mode had a significant effect on percent seagrass cover; overall percent cover was generally lower (˜20%) in procedural controls (which had borders) than in controls. By contrast, Rasheed (Rasheed 1999, 2004), who developed the technique, did not find any effect of borders on seagrass percent cover. One explanation for the difference between Rasheed's work and ours is border size. Rasheed's borders were larger than ours (0.25 m^2^ vs. 0.07 m^2^), meaning that our treatments had a higher edge to area ratio, and as edges are where disturbances occur (e.g., breaking of rhizomes) due to border insertion, there is likely to have been greater overall levels of disturbance caused by borders in our study (Macreadie et al. 2009, 2010). Furthermore, small-sized borders can cause sediment compression, which can alter sediment chemistry by physically forcing nutrients to flux out of the sediment porewater and into the water column (Macreadie et al. 2006).

## Conclusions

Overall, this study suggests that *Zostera muelleri* in Lake Macquarie uses clonal growth as a means of rapidly recovering from small-scale disturbances, and that sexual recovery from seeds play little to no part in recovery at small spatial scales.
